# New Appliances and Things Medical

**Published:** 1900-03-17

**Authors:** 


### NEW APPLIANCES AND THINGS MEDICAL.

**[We shall be glad to receive, at our Office, 28 & 29, Southampton Street, Strand, London, W.C., from the manufacturers, specimens of all new preparations and appliances which may be brought out from time to time.]**

### TURTLE SOUP.

(T. K. Bellis, 15, Bury St., St. Mary Axe, London, E.C.)

As all the world knows, turtle soup is highly nutritious, and doubtless it would long since have become just as much an essential factor in the treatment of phthisis and other wasting diseases as the traditional cod liver oil were it not for the prohibitive price, which has rendered it a luxury even for the rich. The firm of T. K. Bellis has, however, done much to dispel the belief that turtle soup must necessarily be expensive. They carry on the trade of turtle importers on such an enormous scale that, combined with careful management, scientific appliances, and skilled workmen, they are able to turn out the prepared soup at a price hitherto unheard of. The soup for the most part is supplied in glass flacons at 7s., and tins at 5s. per pint. Two pints of this soup if added to an equal quantity of good clear stock will provide for twelve persons a turtle soup as excellent as can possibly be made. Smaller quantities in suitable flacons are supplied especially for invalids in the form of essence or jelly. Jars containing this cost 2s. 6d., and whether cold in the form of a jelly or diluted and converted into soup constitute a most valuable addition to the invalid's dietary.

### SPECIAL SCOTCH WHISKY.

(James Martin and Co., Leith.)

We have received a sample of special liqueur whisky from the above firm. It is a spirit of considerable mellowness and agreeable flavour. It is claimed to be a blend of the finest and oldest Highland whiskies, and from its most excellent qualities we confidently recommend it to the notice of those who either from necessity or choice make whisky their usual form of alcoholic beverage.

